# Factors Affecting Delayed Presentation and Diagnosis of Breast Cancer in Asian Developing Countries Women: A Systematic Review

**DOI:** 10.31557/APJCP.2021.22.10.3081

**Published:** 2021-10

**Authors:** Farida Briani Sobri, Adang Bachtiar, Sonar Soni Panigoro, Dumilah Ayuningtyas, Hardya Gustada, Patria Wardana Yuswar, Aqsha Azhary Nur, R. Cita Resti Anantia Putri, Anggindita Diah Widihidayati

**Affiliations:** 1 *Faculty of Public Health, Universitas Indonesia, Depok, Indonesia. *; 2 *Department of Health Policy and Administration, Faculty of Public Health, Universitas Indonesia, Depok, Indonesia. *; 3 *Department of Surgery, Faculty of Medicine, Universitas Indonesia, Jakarta, Indonesia. *; 4 *Pondok Indah Hospital, Jakarta, Indonesia. *; 5 *Faculty of Medicine Universitas Indonesia, Jakarta, Indonesia. *; 6 *Faculty of Medicine, Universitas Airlangga, Surabaya, Indonesia. *

**Keywords:** Asian developing countries, breast cancer, delayed diagnosis, delayed presentation, examination, knowledge

## Abstract

**Background:**

Advance in screening strategies and management had steadily decreased the mortality rates of breast cancer. In developing countries, conducting screening and early diagnosis of breast cancers may face several problems. This systematic review aims to determine factors affecting the delayed diagnosis of breast cancer in developing countries in Asia.

**Methods:**

Literature research was conducted through Pubmed, ScienceDirect, Scopus, EbscoHost, Cochrane Library, and Google Scholar. The main keywords were “breast cancer”, “delayed diagnosis” and “developing countries”. Both quantitative and qualitative studies were included.

**Results:**

A total of 26 studies were included. The definition of delayed presentation or diagnosis varied from 1 month to 6 months. Among all the factors from patients and providers, breast symptoms and examinations consistently showed a significant contribution in reducing delayed diagnosis. Strengthened by qualitative studies, patients’ knowledge and perception also had a major role in delayed diagnosis.

**Conclusion:**

Among Asian developing countries, breast symptoms and examination, as well as individual knowledge and perception, are the main factors related to delayed diagnosis of breast cancer.

## Introduction

Breast cancer is the second most common type of cancer globally, after lung cancer (Bray et al., 2018). From nearly 10 million deaths due to cancer worldwide, 6.6% are attributed to breast cancer. Significantly higher mortality-incidence ratios and lower survival rates of breast cancer in low- and middle-income countries (LMIC) or developing countries have been highlighted (Panieri, 2012; da Costa Vieira et al., 2017; Rivera-Franco and Leon-Rodriguez, 2018; Francies et al., 2020).

In the last few decades, advances in screening strategies and management have steadily decreased the mortality rates of breast cancer (Kohler et al., 2015). Screening and early diagnosis hold vital roles as the best survival rates are seen in early-stage breast cancers (Richards et al., 1999). Late diagnosis leads to a longer time interval to treatment (surgery or adjuvant therapies), ultimately worsening the overall survival and recurrence rates (Bleicher et al., 2016; Eaglehouse et al., 2019). 

In developing countries, conducting screening and early diagnosis may face hindrance from two main groups of factors. Patient factor groups such as socioeconomic background, family support, social stigma, and culture may influence the patients’ decision to seek professional health care (Norsa’adah et al.; Iskandarsyah et al.; Poum et al.; Huo et al.; Roy et al., 2015; Kumar et al., 2019; Zhang et al.). On the other hand, affordability of service, quality of workforces and resources, compliance to available clinical guidelines in practice, and referral system are health care factors that contribute equally to patient factors (Sacerdote et al., 2013; Kumar et al., 2019; Zhang et al., 2019; Ho et al., 2020; Songiso et al., 2020).

Even though multiple factors affecting delayed diagnosis in Asian developing countries have been studied, the results are heterogeneous and indecisive. Therefore, we conducted a systematic review to determine factors affecting breast cancer delayed diagnosis in Asian developing countries. We specifically chose Asia due to a common shared historical and cultural root in the region, hence avoiding overly diverse results. 

## Materials and Methods

*Search strategy and selection criteria*

This study followed MOOSE guidelines for Meta-Analyses and Systematic Reviews of Observational Studies. Literature reviews were done by multiple investigators (FB, RCRAP, HG, and ADW). These reviews were conducted until November 14, 2020, on six electronic databases: Pubmed, ScienceDirect, Scopus, EbscoHost, Cochrane Library, and Google Scholar. Principally, the main keywords used were, “breast cancer”, “delayed diagnosis”, and “developing countries” (complete search strategies are listed in Supplementary Table 1). The literature that was included was in English and Indonesian languages. We also conducted manual searches for articles that were found during literature reviews. Unpublished studies or conference abstracts were not included in the search. 

*Selection of studies*

Both quantitative and qualitative studies were included. Eligible studies that were included in this systematic review were based on the following: population, intervention, comparison, and outcome (PICO) criteria. The defined population was women over the age of 18 with breast cancer, who lived in Asian developing countries. Interventions were defined as risk factors (naturally existing conditions). The comparison was no risk factors. The outcome was the stage of cancer (locally advanced/advanced stage vs. early stage). Studies with cancer in general, as a topic, earlier than 2010, or without available full text were excluded.

Identified articles from all databases were screened for duplication. Screening based on title and abstract were done and articles that fulfilled exclusion criteria were further excluded. In the end, eligible articles were included in a qualitative synthesis. The whole selection process was conducted by three independent investigators (FBS, RCRAP, and HG). Disagreements between reviewers were reconciled through discussion. Another independent reviewer was sought if an agreement was not achieved.

*Quality assessment and data extraction*

Two independent reviewers (FBS and HG) assessed the risk of bias using the Newcastle-Ottawa Scale (NOS) (Wells et al., 2014). Seven items were assessed for risk of bias in each included study. Every item gave a certain quantity of stars. Studies with a total of ≥7 stars were considered of having a low risk of bias and vice versa. In this systematic review, stars in the instrument were represented by numbers. 

For data extraction, three independent reviewers (FBS, RCRAP, and HG) were involved. Disagreements between reviewers were reconciled by discussion. Another independent reviewer was sought if an agreement was not achieved. Confirmation to the corresponding author was done if there were incomplete or ambiguous data. The extracted data included age at the time of diagnosis, education, economic status, residential status, marital status, age at first birth, parity, post-menopause, breast symptoms, the regularity of self and clinical-breast examination, self and family history of breast disease, knowledge and perception of breast cancer, type of payment, first consultation provider, chances of consultation, false-negative diagnostic test, and alternative therapy or traditional medicine. Other relevant factors were also extracted. Endpoint statistical measures included were odds ratio (OR), relative risk (RR), mortality rate, survival rate, proportion, and p-value for quantitative studies and description of causal factors in qualitative studies. If OR was not stated in the studies, manual calculations were done. If the risk comparison between numerator and denominator was different, the OR was recalculated for adjustment.

*Data synthesis*

If there was adequate data after compilation and analysis, all the data was synthesized into a meta-analysis. A fixed-effect model was used for relatively homogeneous data, while a random-effect model was used for heterogeneous data. Test of heterogeneity is using I^2 ^statistics and Cochran’s Q test. A funnel plot analysis is used to investigate the possibility of any publication bias in meta-analysis. No predefined subgroup analysis were planned.

## Results

**Figure 1:**
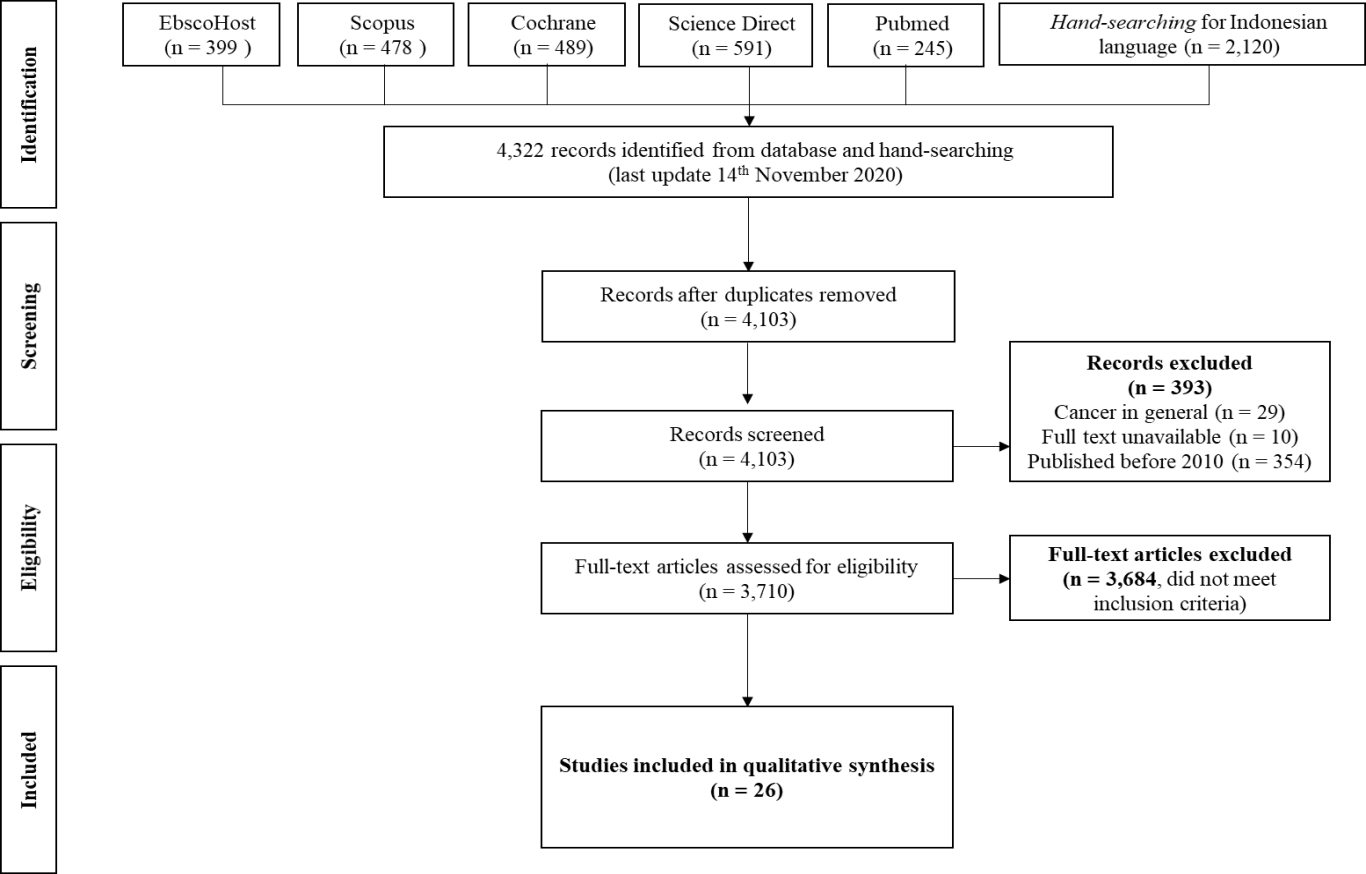
PRISMA Diagram of the Study

A total of 4,322 studies were identified at the beginning and after a thorough process (shown in Figure 1), 26 studies were included (shown in Table 1). None of the included studies had incomplete information so further confirmation to the author was not required. Further details on the steps of the study can be reviewed in Supplementary Table 1. Details of excluded studies were in Supplementary Table 2. 

Most of the studies were conducted in Malaysia and India. The definitions of delayed diagnosis varied from 1 month to 6 months (shown in Table 2). From 14 quantitative studies, 12 were cross-sectional studies and the other two were observational ones. Out of 12 cross-sectional studies, 5 of them had a high risk of bias, while the rest had a low risk of bias (shown in Table 3). Factors related to delayed diagnosis were summarized into socioeconomic status, maternity status, breast symptoms and examinations, healthcare-related, patients’ knowledge and perceptions, and other factors as seen in Table 4. None of those factors were found to be strongly related to delayed diagnosis of breast cancer, but breast symptoms and examinations, and patient knowledge and perception, were major contributors. Results from qualitative studies, as shown in Table 5, validate the information on how different those factors were between the various study settings.

Meta-analysis was not possible due to the varying definitions of the delayed diagnosis (some studies used three months as a cutoff, while others used six months or more), uneven distribution of the location of the studies (mainly in Southeast Asia and South Asia), and high risk of bias in the majority of the studies.

**Table 1:** List of Included Studies and Database

No	First Author	Publication Years	Title	Journal	Database
1	Chintamani	2011	Patient and provider delays in breast cancer patients attending a tertiary care centre: a prospective study	J R Soc Med Sh Rep	Hand Searching
2	Bachok Norsa’adah	2011	Diagnosis delay of breast cancer and its associated factors in Malaysian women	BMC Cancer	PubMed
3	Nur Aishah Taib	2011	Recognizing symptoms of breast cancer as a reason for delayed presentation in Asian women – the psycho-socio-cultural model for breast symptom appraisal: opportunities for intervention	Asian Pac J Cancer Prev	PubMed
4	Bachok Norsa’adah	2012	Understanding barriers to Malaysian women with breast cancer seeking help	Asian Pac J Cancer Prev	PubMed
5	Sumarni Mohd Ghazali	2013	Non-practice of breast self examination and marital status are associated with delayed presentation with breast cancer	Asian Pac J Cancer Prev	PubMed
6	Srikanthi Lakshmi Bodapati	2013	Oncologist perspective on breast cancer screening in India- results from a qualitative study in Andhra Pradesh	Asian Pac J Cancer Prev	PubMed
7	Nur Aishah Taib	2013	A grounded explanation of why women present with advanced breast cancer	World J Surg	EbscoHost
8	Ario Djatmiko	2013	Profil cancer delay pada kasus kanker payudara di RS Onkologi Surabaya	Indonesian Journal of Cancer	Hand Searching
9	Amornsak Poum	2014	Factors associated with delayed diagnosis of breast cancer in Northeast Thailand	J Epidemiol	PubMed
10	Aulia Iskandarsyah	2014	Psychosocial and cultural reasons for delay in seeking help and nonadherence to treatment in Indonesian women with breast cancer: a qualitative study	Health Psychology	Hand Searching
11	Aulia Iskandarsyah	2014	Consulting a traditional healer and negative illness perceptions are associated with non-adherence to treatment in Indonesian women with breast cancer	Psycho-Oncology	Hand Searching
12	Sedigheh Pakseresht	2014	Stage at diagnosis and delay in seeking medical care among women with breast cancer, Delhi, India	Iran Red Crescent Med J	Hand Searching
13	Bimal Roy	2015	Pattern of delayed presentation of breast cancer patients: evidence from Rangpur Medical College Hospital, Rangpur, Bangladesh	Advances in Cancer Research and Therapy	Hand Searching
14	Qiang Huo	2015	Delay in diagnosis and treatment of symptomatic breast cancer in China	Ann Surg Oncol	PubMed
15	Namrata Thakur	2015	Delay in presentation to the hospital and factors affecting it in breast cancer patients attending tertiary care center in Central India	Indian J Cancer	EbscoHost
16	Jennifer NW Lim	2015	Barriers to early presentation of self-discovered breast cancer in Singapore and Malaysia: a qualitative multicenter study	BMJ Open	PubMed
17	Gusti Ayu Resa Dyanti	2016	Faktor-faktor keterlambatan penderita kanker payudara dalam melakukan pemeriksaan awal ke pelayanan Kesehatan	Jurnal Kesehatan Masyarakat	Hand Searching
18	Noor Mastura Mohd Mujar	2017	Complementary and alternative medicine (CAM) use and delays in presentation and diagnosis of breast cancer patients in public hospitals in Malaysia	Plos One	Scopus
19	Khurseda Akhtar	2018	Use of alternative medicine is delaying health-seeking behavior by Bangladeshi breast cancer patients	Eur J Breast Helath	Hand Searching
20	Safira Dhia Rahmawaty	2019	Hubungan factor-faktor treatment delay dengan kasus kanker payudara stadium lanjut di RSUD Abdul Wahab Sjahranie Samarinda tahun 2019	Jurnal Psikologi	Hand Searching
21	Arvind Kumar	2019	Delays in diagnosis and treatment of breast cancer and the pathways of care: a mixed methods study from a tertiary cancer centre in North East India	Asian Pac J Cancer Prev	PubMed
22	Huaguo Zhang	2019	Patient delay and associated factors among Chinese women with breast cancer: a cross-sectional study	Medicine	PubMed
23	Mehreen Baig	2019	Factors influencing delayed presentation of breast cancer at a tertiary care hospital in Pakistan	Cancer Reports	PubMed
24	Uzma Shamsi	2020	Patient delay in breast cancer diagnosis in two hospitals in Karachi, Pakistan: preventive and life-saving measures needed	JCO Global Oncol	Hand Searching
25	Shivaraj Nailur Somana	2020	Time interval between self-detection of symptoms to treatment of breast cancer	Asian Pac J Cancer Prev	Hand Searching
26	Solikhah Solikhah	2020	Breast cancer stigma among Indonesian women: a case study of breast cancer patients	BMC Women’s Health	EbscoHost

**Table 2:** Characteristics of Included Studies

No	First Author	Publication Year	Country	Study Design	Number of Participants	Definition of Delayed Diagnosis	Affecting Factors
1	Chintamani	2011	India	Qualitative study	100	-	(1) Illiteracy and lack of adequate healthcare services (2) Unregistered medical practitioners or quacks
2	Bachok Norsa'adah	2011	Malaysia	Cross-Sectional study	328	The recognition of symptoms to the histological diagnosis was more than 6 months	(1) The use of alternative therapy (2) Breast ulcer (3) Palpable axillary lymph nodes (4) False-negative diagnostic test (5) Non-cancer interpretation (6) A negative attitude toward treatment
3	Nur Aishah Taib	2011	Malaysia	Qualitative study	19	-	(1) Symptom knowledge (no recognition of breast lump and non-lump symptoms) (2) Lacked confidence in breast changes (3) A strong belief that family history should be present for someone to be at risk of cancer (4) Misled breast changes due to pregnancy (5) Misdiagnosed by the doctor in charge (6) The misconception of the disease progression (7) Denial (8) Lack of support from significant others
4	Bachok Norsa'adah	2012	Malaysia	Qualitative study	12	-	(1) Poor knowledge or awareness of breast cancer (2) Fear of cancer consequences (3) Beliefs in complementary alternative medicine (4) Sanction by others (5) Other priorities (6) Denial of disease (7) An attitude of wait and see (8) Health care system weakness
5	Sumarni Mohd Ghazali	2013	Malaysia	Cross- sectional study	250	Delayed presentation: presenting to a physician >3 months after self-discovery of a symptom	(1) Divorced or widowed women (2) Women who never performed breast self-examination
6	Srikanthi Lakshmi Bodapati	2013	India	Qualitative study	10	-	(1) Fear (2) Embarrassment (3) Cost (4) Ignorance (5) Negligence (6) Easy going attitude
7	Nur Aishah Taib	2013	Malaysia	Qualitative study	19	-	(1) Appraisal delay (2) Disclosure delay (3) Illness delay (4) Behavioral/referral delay (5) Scheduling delay (6) Diagnostic delay (7) Treatment decision delay (8) Treatment delay
8	Ario Djatmiko	2013	Indonesia	Qualitative study	152	Patient delay: Late > 3 months Referral delay: > 4 weeks	(1) Lack of knowledge about the danger of breast lump (2) Fear
9	Amornsak Poum	2014	Thailand	Cross-sectional study	180	Patient delay: Time from onset of symptoms to first consultation with a health care provider Doctor delay: Time from the first consultation with a health care provider to the diagnosis of breast cancer	Patient delay: (1) Higher family income (2) Smoking Doctor delay: (1) Age at first birth (2) Previous breast symptoms (3) Number of consultations with a surgeon before diagnosis Regarding stage of breast cancer: (1) Age at diagnosis (2) Education (3) Family income (4) Time to referral (5) Number of consultations with a surgeon before diagnosis
10	Aulia Iskandarsyah	2014	Indonesia	Qualitative study	50	The period from the first breast symptom to the first medical consultation > 3 months	(1) Lack of awareness and knowledge (2) Cancer beliefs (3) Treatment beliefs (4) Financial problems (5) Emotional burden (6) Severe side effects (7) Paternalistic style of communication (8) Unmet information needs
11	Aulia Iskandarsyah	2014	Indonesia	Observational study	70	-	(1) Consulting a traditional healer before diagnosis (2) More negative illness perceptions
12	Sedigheh Pakseresht	2014	India	Cross-sectional study	172	-	Lower income
13	Bimal Roy	2015	Bangladesh	Cross-sectional study	62	Time intervals of > 12 weeks from first symptom recognition to first medical consultation and final diagnosis and treatment	(1) Age >50 years (2) Low socioeconomic status (3) Low education level (4) Homeopath or other local medication
No	First Author	Publication Year	Country	Study Design	Number of Participants	Definition of Delayed Diagnosis	Affecting Factors
14	Qiang Huo	2015	China	Cross-sectional study	1431	Time from initial symptom to breast cancer diagnosis of 1 month (30 days)	(1) Patient residential status (2) Initial symptom (3) Menopausal status (4) History of breast disease
15	Namrata Thakur	2015	India	Qualitative study	120	The time lag since self-detection of a lump in the breast and presentation to any health facility	(1) Women living in a rural area (2) Lower socioeconomic status (3) Older age
16	Jennifer NW Lim	2015	Multicenter (Malaysia & Singapore)	Qualitative study	67	-	(1) The poor quality of online website information about breast symptoms (2) Financial issues (3) The negative influence of relatives (4) Perceived poor quality of care and services in state-run hospitals and misdiagnosis by healthcare professionals
17	Gusti Ayu Resa Dyanti	2016	Indonesia	Qualitative study	108	-	(1) Level of education (2) Level of knowledge (3) Financial (4) Availability of information (5) Husband/ family support (6) Early detection behavior
18	Noor Mastura Mohd Mujar	2017	Malaysia	Cross-sectional study	340	Presentation delay: Time from symptom discovery to the first presentation >3 months Diagnosis delay: Time from the first presentation to the diagnosis >1 month	(1) Complementary alternative medicine (CAM) use (2) Symptoms without breast lumps
19	Khurseda Akhtar	2018	Bangladesh	Cross-sectional study	200	Total delay or delay: The period between a woman first noticing a breast cancer symptom and receiving treatment for this can be referred to as delay or total delay Provider delay: Refers to the period between the initial medical consultation and definitive treatment of the cancer Patient delay: The period that will be used is the time from discovered the breast symptom to the time a woman seeks evaluation of the symptom by a health care provider Health care provider: Defined as a person, seek medical consultation from the first detection of breast symptom(s) to diagnosis and treatment	(1) The use of alternative medicine homeopathy (2) Residence (3) Patients perceptions (4) Less amount of money required (5) The lump would be small (6) Duration of local treatment used
20	Safira Dhia Rahmawaty	2019	Indonesia	Qualitative study	97	Patient delay: See medical attention > 3 months	(1) Fear of diagnosed with cancer (2) Use of alternative medicine
21	Arvind Kumar	2019	India	Cross-sectional study	269	Presentation delay: Recognition of symptoms to first provider consultation Delay: >3 months following symptom recognition	(1) Misconception about the disease (2) Perceived stigma (3) Fear (4) Denial of cancer (5) Attribution of symptoms to trivial conditions (6) Family responsibilities (7) The embarrassment of breast examination by a male doctor
22	Huaguo Zhang	2019	China	Cross-sectional study	283	Patients waited ≥90 days to access medical treatment after symptom onset	(1) Knowledge of breast cancer symptoms (2) External health locus of control (3) Breast self-examination or physical examination (4) Perceived health competence (5) Family support (6) Pain stimulation (7) Age
23	Mehreen Baig	2019	Pakistan	Observational study	89	-	(1) Lack of knowledge about breast cancer (2) Lack of availability of health care services (3) Purdah and religious reasons (4) Fear of being diagnosed with cancer (5) Alternative treatment
24	Uzma Shamsi	2020	Pakistan	Cross-sectional study	499	The patient sought medical help >1 month after noticing possible symptoms of breast cancer Delay was further categorized into “intermediate delay” (<3 months) and “long delay” (≥3 months).	(1) Lack of awareness about the significance of the lump (2) Using complementary and alternative medicine and traditional treatment (3) Presented to a health care provider with a breast lump but no action was taken (4) Wrongly reassured about the lump without mammography or biopsy (5) Anxiety (6) Fears (7) Misconceptions regarding diagnosis and treatment (8) Possible adverse effects on their relationship with their husband
No	First Author	Publication Year	Country	Study Design	Number of Participants	Definition of Delayed Diagnosis	Affecting Factors
25	Shivaraj Nallur Somana	2020	India	Cross-sectional study	181	-	Delay in seeking medical care: (1) Lack of awareness in identifying the breast cancer symptoms (2) Assuming that the symptom would resolve by itself (3) Absence of pain (4) Changes in the body attributed to common illness Delay in getting a definitive diagnosis at tertiary care hospitals: (1) Visits to multiple medical practitioners who did not suspect cancer Delay in seeking treatment after diagnosis at tertiary care: (1) Fear of treatment (2) Financial dependence on the family (3) Disfiguring of the body (4) The stigma attached with the disease (5) Long treatment procedure
26	Solikhah Solikhah	2020	Indonesia	Qualitative study	8	-	(1) Embarrassment (2) Traditional healing practice which is known as ‘kerokan’ (involves scraping of the skin) and consumption of a traditional drink (3) Financial difficulties

**Table 3:** Assessment of Risk of Bias of the Quantitative Study (Cross-Sectional)

No	Study	Selection				Comparability	Outcome		Risk of Bias
		RS	SS	NR	AE	(C)	AO	ST	
1	Norsa’adah, 2011	1	1	0	2	2	2	1	9 (Low)
2	Ghazali, 2013	1	1	1	1	2	1	1	8 (Low)
3	Mujar, 2013	1	1	0	2	2	2	1	9 (Low)
4	Poum, 2014	0	0	1	0	1	2	1	5 (High)
5	Pakseresht, 2014	1	0	0	2	0	2	0	5 (High)
6	Roy, 2015	0	0	1	1	0	1	0	3 (High)
7	Huo, 2015	1	0	0	1	2	2	1	7 (Low)
8	Akhtar, 2018	1	1	0	1	0	1	1	5 (High)
9	Kumar, 2019	1	1	1	1	2	2	0	8 (Low)
10	Zhang, 2019	1	1	0	2	1	2	1	8 (Low)
11	Shamsi, 2020	1	1	0	2	2	2	1	9 (Low)
12	Somana, 2020	1	1	0	2	0	2	0	6 (High)

* 1 representing 1 star; RS, Representativeness of the samples; SS, sample size; NR, non-respondents; AE, ascertainment of the exposure; C, comparability; AO, assessment of the outcome; ST, statistical test

**Table 4:** Factors Affecting Delayed Diagnosis of Breast Cancers Based on Included Quantitative Studies

Factors associated	Author, year		OR (95%CI)		p-value		Risk of bias		
Socioeconomic status									
Age at diagnosis									
>45 years	Kumar et al, 2019		OR 1.21		0.468		Low		
			(0.72-2.06)						
	Somanna et al, 2019		OR 0.69				High		
			(0.38-1.28)						
>50 years	Mujar et al, 2017		OR 1.21		0.47		Low		
			(0.72-2.02)						
	Parakseresht et al, 2014		OR 1.09		0.784		High		
			(0.58-2.04)						
	Roy et al, 2015		OR 12.57		0.013		High		
			(3.63-43.58)						
Age (numeric)	Zhang et al, 2019		OR 1.03		0.049		Low		
			(1.00-1.06)						
	Akhtar et al, 2018		OR 0.84		0.589		High		
			(0.45-1.58)						
Formal Education									
High education	Kumar et al, 2019		OR 0.53		0.07		Low		
			(0.26-1.07)						
	Roy et al, 2015		OR 0.06		0.018		High		
			(0.01-0.37)						
	Akhtar et al, 2015		OR 0.83		0.551		High		
			(0.44-1.55)						
Moderate education	Kumar et al, 2019		OR 0.77		0.82		Low		
			(0.41-1.44)						
	Mujar et al, 2017		OR 0.98		0.959		Low		
			(0.48-1.98)						
	Roy et al, 2015		OR 0.60		0.478		High		
			(0.15-2.46)						
Low education	Kumar et al, 2019		OR 1.07		0.145		Low		
			(0.45-2.50)						
	Mujar et al, 2017		OR 2.01		0.185		Low		
			(0.71-5.65)						
Economic status									
Moderate income vs low income	Poum et al, 2014		OR 2.83		0.04		High		
			(1.01-7.95)						
	Roy et al, 2015		OR 0.05		<0.01		High		
			(0.01-0.25)						
	Somanna et al, 2019		OR 1.17		0.746		High		
			(0.44-3.08)						
High income vs low income	Mujar et al, 2017		OR 0.64		0.108		Low		
			(0.37-1.10)						
	Roy et al, 2015		OR 0.08		0.03		High		
			(0.01-0.85)						
	Shamsi et al, 2020		OR 0.38				Low		
			(0.13-1.11)						
Unemployed	Mujar et al, 2017		OR 0.68		0.174		Low		
			(0.40-1.17)						
	Akhtar et al, 2018		OR 0.93		0.859		High		
			(0.40-2.13)						
	Parakseresht et al, 2014		OR 0.96		0.958		High		
			(0.22-4.17)						
			OR (95%CI)		p-value		Risk of bias		
Residential status								
Urban vs Rural	Huo et al, 2015		OR 0.71		0.004		Low	
			(0.56-0.89)					
	Parakseresht et al, 2014		OR 0.54		0.057		High	
			(0.29-1.02)					
	Roy et al, 2015		OR 0.68		0.758		High	
			(0.20-2.33)					
	Akhtar et al, 2018		OR 0.72		0.309		High	
			(0.38-1.35)					
	Somanna et al, 2019		OR 1.18		0.582		High	
			(0.65-2.18)					
Marital status								
Unmarried	Ghazali et al, 2013		OR 1.93		0.229		Low	
			(0.66-5.64)					
	Muhar et al, 2017		OR 1.27		0.387		Low	
			(0.73-2.22)					
	Pakseresht et al, 2014		OR 1.75		0.12		High	
			(0.87-3.53)					
	Roy et al, 2015		OR 0.81		0.826		High	
			(0.12-5.42)					
	Akhtar et al, 2018		OR 1.11		0.778		High	
			(0.53-2.34)					
Divorced/Widowed	Ghazali et al, 2013		OR 2.67		0.008		Low	
			(1.30-5.50)					
	Roy et al, 2015		OR 1.51		0.501		High	
			(0.45-5.06)					
Age at first birth								
>20	Poum et al, 2014		OR 0.29		0.003		High	
			(0.13-0.65)					
21-25 years old	Kumar et al, 2019		OR 0.36		0.001		Low	
			(0.19-0.66)					
25-38 years old	Kumar et al, 2019		OR 1.08		0.839		Low	
			(0.53-2.21)					
Parity								
Less than 3 delivery	Poum et al, 2014		OR 0.92		0.85		High	
			(0.41-2.05)					
	Parakseresht et al, 2014		OR 1.27		0.479		HIgh	
			(0.65-2.48)					
Postmenopause	Huo et al, 2015		OR 0.79		0.026		Low	
			(0.63-0.97)					
Breast symptoms and examination								
Breast symptoms								
Presence of any breast symptoms	Poum et al, 2014		OR 0.36		0.01		High	
			(0.16-0.80)					
	Mujar et al, 2017		OR 0.46		0.033		Low	
			(0.22-0.95)					
	Akhtar et al, 2018		OR 1.12		0.774		High	
			(0.51-2.46)					
Nipple discharge (vs Breast mass)	Huo et al, 2015		OR 0.32		0.007		Low	
			(0.14-0.73)					
Breast ulcer	Norsa'adah et al, 2011		OR 0.17		0.008		Low	
			(0.04-0.62)					
Pain	Zhang et al, 2019		OR 0.19 (0.05-0.79)		0.023		Low	
Factors associated	Author, year		OR (95%CI)		p-value		Risk of bias	
Breast self-examination								
Never		Ghazali et al, 2013		OR 2.19		0.028		Low
				(1.09-4.38)				
Irregular		Ghazali et al, 2013		OR 1.18		0.686		Low
				(0.53-2.64)				
Clinical Breast Examination		Zhang et al, 2019		OR 0.06		0.015		Low
				(0.01-0.59)				
		Norsa'adah et al, 2011		OR 0.45		0.008		Low
				(0.25-0.81)				
History of breast disease		Huo et al, 2015		OR 0.70 (0.49-1.01)		0.058		Low
Family history with breast cancer		Mujar et al, 2017		OR 1.25		0.486		Low
				(0.68-2.27)				
Healthcare-related factors								
Healthcare accessibility								
Low utilization		Akhtar et al, 2018		OR 1.73		0.15		High
				(0.82-3.64)				
Long distance (>2 km)		Somanna et al, 2019		OR 1.03		0.941		High
				(0.52-2.04)				
Long distance (>10 km)		Kumar et al, 2019		OR 0.93		0.814		Low
				(0.49-1.75)				
Long traveling time (>60 min)		Poum et al, 2014		OR 2.66		0.01		High
				(1.17-6.04)				
Self-payment (vs insurance)		Poum et al, 2014		OR 1.42		0.64		High
				(0.32-6.24)				
		Somanna et al, 2019		OR 0.51		0.424		High
				(0.10-2.62)				
First consultation to nonphysician (vs physician)		Poum et al, 2014		OR 1.39		0.69		High
				(0.26-7.21)				
Physician consult >2 times to surgeon		Poum et al, 2014		OR 2.93 (1.33-6.44)		0.007		High
False-negative diagnostic test		Norsa'adah et al, 2011		OR 5.32		<0.001		Low
				(2.32-12.21)				
Alternative therapy/ traditional medicine		Norsa'adah et al, 2011		OR 1.77		0.029		Low
				(1.06-2.94)				
		Mujar et al, 2017		OR 2.58		<0.001		Low
				(1.59-4.17)				
		Roy et al, 2015		OR 8.25		0.012		High
				(2.52-27.16)				
		Akhtar et al, 2015		OR 4.35		<0.001		High
				(2.21-8.59)				
Patient knowledge and perception								
Good literacy on breast cancer symptoms		Zhang et al, 2019		OR 0.72		<0.01		Low
				(0.64-0.80)				
		Pakseresht et al, 2014		OR 0.81		0.506		High
				(0.43-1.52)				
		Somanna et al, 2019		OR 0.49		0.03		High
				(0.26-0.93)				
Patient good perception		Akhtar et al, 2018		OR 0.43		0.298		High
				(0.09-2.10)				
		Zhang et al, 2019		OR 0.87		<0.01		Low
				(0.81-0.94)				
Negative attitude		Norsa'adah et al, 2011		OR 2.09		0.016		Low
Factors associated		Author, year		OR (95%CI)		p-value		Risk of bias
Mental upset		Akhtar et al, 2018		OR 0.78		0.5		High
				(0.36-1.65)				
Other factors								
Smoking status								
Current smoker		Kumar et al, 2019		OR 1.54		0.215		Low
				(0.78-3.05)				
Non-smoker		Poum et al, 2014		OR 0.15		0.06		High
				(0.02-1.03)				
Sufficient physical activity		Kumar et al, 2019		OR 0.44		0.01		Low
				(0.23-0.83)				
Family support		Zhang et al, 2019		OR 0.91		0.013		Low
				(0.84-0.98)				
		Akhtar et al, 2018		OR 3.37		<0.01		High
				(1.65-6.89)				

**Table 5:** Summary of the Qualitative Studies and Other Types of Studies

No	Titles	First Author	Year	Type of Study	Summary	Factors
1	Patient and provider delays in breast cancer patients attending a tertiary care center: a prospective study	Chintamani	2011	Qualitative Study	100 patients India	Non-competent health workers Patients illiteracy
2	Recognizing symptoms of breast cancer as a reason for delayed presentation in Asian women--the psycho-socio-cultural model for breast symptom appraisal: opportunities for intervention	Nur Aishah Taib	2011	Qualitative Study	19 patients Malaysia	Symptoms recognition Knowledge of disease and its outcome
3	Understanding barriers to Malaysian women with breast cancer seeking help	Bachok Norsa’adah	2012	Qualitative Study	12 patients Malaysia	Poor knowledge and attitude Fear of cancer consequences Alternative medicine Social stigma Denial Health care system weakness
4	Oncologist perspectives on breast cancer screening in India- results from a qualitative study in Andhra Pradesh	Srikanthi Lakshmi Bodapati	2013	Qualitative Study	Subjects are oncologists India	Awareness Rural Poor socioeconomic status Fear Embarrassment Cost Ignorance Easy going attitude
5	A grounded explanation of why women present with advanced breast cancer	Nur Aishah Taib	2013	Qualitative Study	4–24 months Malaysia	Knowledge of disease and treatment Psychological and physical resources and support
6	Profil cancer delay pada kasus kanker payudara di RS Onkologi Surabaya	Ario Djatmiko	2013	Qualitative Study	55 patients Indonesia	Knowledge of symptoms Alternative therapy
7	Psychosocial and cultural reasons for delay in seeking help and nonadherence to treatment in Indonesian women with breast cancer: a qualitative study	Aulia Iskandarsyah	2014	Qualitative Study	50 patients Indonesia	Lack of awareness and knowledge Cancer beliefs Treatment beliefs Financial problems Emotional burden Severe side effects Paternalistic style of communication Unmet information needs
8	Consulting a traditional healer and negative illness perceptions are associated with non-adherence to treatment in Indonesian women with breast cancer	Aulia Iskandarsyah	2014	Observational Study	70 patients Indonesia	Consulting a traditional healer before diagnosis More negative illness perceptions
9	Delay in presentation to the hospital and factors affecting it in breast cancer patients attending tertiary care center in Central India	Namrata Thakur	2015	Qualitative Study	120 patients India median delay: 6 months (2 days–6 years)	Structural à poor health facilities, distance, no work off-time. Organizational à complex health system, interaction with medical staff Psycho-socio-cultural à poor motivation, denial, treatment mistrust, fear of family burden. Traditional medicines
10	Barriers to early presentation of self-discovered breast cancer in Singapore and Malaysia: a qualitative multicentre study	Jennifer NW Lim	2015	Qualitative Study	67 patients Singapore and Malaysia	Symptom misinterpretation by patients and healthcare Fear of diagnosis and treatment due to fatalistic view of ‘cancer’ and poor Knowledge of treatment Fear of hospitalization Denial Preference for alternative and traditional medicine Financial issue Misinformed by relatives Cultural (stigma) and marriage issues Fated because of a family history of breast cancer
11	Faktor – faktor keterlambatan penderita kanker payudara dalam melakukan pemeriksaan awal ke pelayanan kesehatan	Gusti Ayu Resa Dyanti	2016	Qualitative Study	108 patients Indonesia	Level of education Knowledge Cost Information Husband/family support Self-breast examination
12	Hubungan faktor – faktor treatment delay dengan kasus kanker payudara stadium lanjut di RSUD Abdul Wahab Sjahranie Samarinda tahun 2019	Safira Dhia Rahmawaty	2019	Qualitative Study	97 patients Indonesia	Fear of cancer Knowledge Alternative therapy
13	Factors influencing delayed presentation of breast cancer at a tertiary care hospital in Pakistan	Mehreen Baig	2019	Observational Study	89 patients Pakistan	Lack of knowledge about breast cancer Lack of availability of health care services Purdah and religious reasons Fear of being diagnosed with cancer Alternative treatment
14	Breast cancer stigma among Indonesian women: a case study of breast cancer patients	Solikhah Solikhah	2020	Qualitative Study	8 patients Indonesia	Fear Social stigma Alternative therapy Financial Knowledge about symptoms

## Discussion

This systematic review was the first one to summarize factors associated with delayed diagnosis of breast cancers in Asian developing countries. Clinicians and stakeholders can use the information gained from this study, to design preemptive models against elements that cause the delay. 

The inclusion of qualitative research strengthened this study by enhancing information obtained through quantitative research. It gave a more unquantified perspective on how patients’ perception, fear of cancer diagnosis, knowledge, and healthcare accessibility played an important role in preventing delayed diagnosis of breast cancer. On the other hand, the weakness of this study was the unfeasibility of conducting a meta-analysis due to the aforementioned reasons.

The risk of publication bias was relatively low because some factors in the included studies were not statistically significant, yet the studies were still published. However, most of the included studies had a high risk of bias, especially in the aspect of patient selection, and thus might have led to selection bias. We hypothesized that the high risk of selection bias was probably caused by the difficulty in collecting accurate pre-diagnosis information from patients. Moreover, the information was collected retrospectively, causing a risk of recall bias. Some studies had a sample size that was insufficient, so the data did not necessarily represent the population. On the other hand, confounder analysis was addressed adequately. Although strength of evidence for each factor related to breast cancer delayed diagnosis are various, we had seen some tendency as will be discussed further in this section. 

Three months as the cut-off to define delayed diagnosis was mostly used in included studies. The previous meta-analysis clarified that globally, a delay (defined as the delay from the onset of symptoms to the start of treatment) of more than three months was strongly related to a poor survival rate (Richards et al., 1999).

Principally, reasons for a delay in diagnosis were grouped into patients’ and providers’ delays. However, we found some factors fell into a “grey area”. For example, a patient’s visit to a non-physician at the time of the onset of symptoms can be influenced by the patient’s perception. However, it also can be caused by the low quality of available health providers or the long distance to the nearest provider. Thus, both factors should be carefully investigated when optimizing the screening and early diagnosis of breast cancers. 

A past systematic review reported that single marital status and advanced age were the only sociodemographic factors that seemed to be strongly associated with patient delay (Richards et al., 1999). However, further studies have been shown to obtain contradictory results (Rivera-Franco and Leon-Rodriguez, 2018). Meanwhile, higher formal education and socioeconomic status could reduce delayed diagnosis, even though several studies, contrastingly, did not report similar results (Bodapati and Babu, 2013; Lim et al., 2015; Roy et al., 2015; Dyanti and Suariyani, 2016; Solikhah et al., 2020). Residential status and distances to healthcare facilities were also among factors related to delayed diagnosis in breast cancer (Bodapati and Babu, 2013; Thakur et al., 2015). Among maternity status, age at first birth significantly reduced delayed diagnosis, but its categorization (>20 years old and 21–25 years old) was not well related to breast cancer’s biomolecular background (Poum et al., 2014; Kumar et al., 2019). Menopausal status that strongly affected delayed diagnosis of breast cancer might be explained by less breast mass density in postmenopausal women which allows for early detection during breast self-examination (BSE) or clinical breast examination (CBE).

In our opinion, the delay from the provider is rooted in two stages. The first stage is primary health care. Ideally, every country should have a breast cancer screening program that is bound by the law to reinforce the screening uptake. Since approximately one-third of Asian countries have been categorized as low- and lower-middle-income countries, screening modalities should be tailored according to the economic capability of the country (World Bank, 2020). One excellent example is the recommendation from World Health Organization (WHO) that such countries may attempt screening by CBE for women aged 50–69 years old if mammography is too expensive and not feasible (World Health Organization, 2014). Moreover, the primary healthcare providers’ CBE skills should be assessed and evaluated periodically. To ensure non-substandard CBE skills, the associated educational institution can aid in the form of hands-on training or other kinds of knowledge-sharing sessions. A clear referral system with geographically accessible secondary or tertiary healthcare facilities is also needed.

The second stage of provider delay is the referral stage. The problem at this stage stems from the inability of secondary healthcare to provide an accurate and quick diagnosis of breast cancer due to a lack of skills or facilities/resources. Therefore, both institutional and healthcare centers must be improved to rectify this problem. An initiative from the government for equal distribution of secondary and tertiary healthcare providers (e.g., general surgeons or surgical oncologists) and healthcare facilities are also required. Overall, while funding systems may initially become a problem in developing countries, a well-executed established system can serve as a driving force for physicians to act according to the clinical guidelines. Eventually, proper and on-time screening and diagnosis can bring down delayed diagnosis cases and overall cost.

The Government’s commitment holds incredible importance in the timely management of cancer in general. As an example, the law in Brazil states that the time from diagnosis to treatment of breast cancer should be less than 60 days (Ferreira et al., 2020). In the United Kingdom, suspected cases of colorectal cancer have to be referred within two weeks (Thomas and Burnet, 2001). While they have flaws, those programs showed the willingness of regulators to be involved in the management of cancer. On the other hand, a system that is too strict and non-adaptive may cause further delays. An example of this is the referral system in Indonesia, which is based on the authors’ experience. The waiting time for diagnosis of individuals suspected to have breast cancer is unnecessarily prolonged due to the inability of patients to be referred directly to a cancer center or similar health facility with definitive diagnostic capabilities, because of administrative reasons from National Health Insurance. This also hampers the physician’s ability to quickly perform the required diagnostic procedures. For instance, a biopsy cannot be performed in the same visit as initial laboratory and radiologic workups due to a “cost package limit”.

An important finding in this systematic review was that the presence of breast symptoms (any symptoms including breast mass, ulcer, or pain and nipple discharge) was consistently related to less delayed diagnosis, both in quantitative and qualitative studies (Norsa’adah et al., 2011; Poum et al., 2014; Huo et al., 2015; Lim et al., 2015). However, because symptomatic breast cancer may be indicative of a more advanced stage of cancer, other factors that can encourage individuals to get diagnosed early should be explored. 

Breast cancer detection through BSE, although not routinely done, was among the most effective methods for early detection of breast cancer (Ghazali et al., 2013; Dyanti and Suariyani, 2016). CBE is also important for the early detection of breast cancers as it significantly reduced delay in diagnosis and presentation (Norsa’adah et al., 2011; Huo et al., 2015). Both of them should be the main strategies in breast cancer detection in developing countries as conducting BSE and CBE do not require many resources. Therefore, health campaigns to educate women to understand the importance of BSE should be done massively and repetitively. Health workers’ skills to perform CBE appropriately, as well as their ability to detect suspicious cancers that need to be referred during CBE, must be nourished and refreshed through workshops and continuous medical educations (CMEs). In short, public health strategies should be developed to optimize both BSE and CBE. 

Patients’ knowledge and perception, or a good literacy on breast cancer, especially about its symptoms, are related to less delayed diagnosis. From qualitative evidence, those factors were known to influence the act of seeking a professional health provider and avoiding alternative therapy, in addition to a less negative attitude toward medical services. Psychosocial factors, such as the perception of fear, denial, embarrassment, ignorance, and emotional burdens were reinforcing factors that need support and motivation from close relatives and the environment (Norsa’adah et al., 2012; Bodapati and Babu, 2013; Iskandarsyah et al., 2014; Huo et al., 2015). In most Asian countries, family bonds along with husbands’ or fathers’ decisions have a strong impact on women’s actions (including health issues); therefore, education on the importance of breast cancer screening and early diagnosis should reach all family members and community members. 

Health service quality is another key factor in the prevention of delayed diagnosis. Still, the interpretation of quality was varied among studies, such as competency of health workers, time and distance to reach healthcare services, and quality of the diagnostic test. In addition, there was a tendency in Asian cultures to visit alternative or traditional medicine (Norsa’adah et al., 2011; Ghazali et al., 2013; Thakur et al., 2015). The intention to visit alternative therapy may have arisen from either personal preferences or due to unsatisfied services received from a previous healthcare provider (Norsa’adah et al., 2012; Thakur et al., 2015; Akhtar et al., 2018). 

Further meta-analyses should be conducted whenever more data are available. On the other hand, high-quality observational studies may be undertaken to obtain specific information on how to optimize breast cancer detection. It ought to have an adequate sample size and consider both patient and provider factors so that the data is more comprehensive. More evidence is needed because different approaches to prevent the delayed diagnosis of breast cancers should be considered in developing countries as compared to high-income countries. Furthermore, to improve patients’ knowledge and perception, every country should conduct individualized anthropological research to discover an appropriate communication, information, and education model which takes into account the local culture.

In conclusion, among Asian developing countries, breast cancer symptoms and examinations, as well as individual knowledge and perception, are the main factors related to delayed diagnosis of breast cancer. Socioeconomic, maternity, and health service quality have various impacts on the delayed diagnosis of breast cancers, and thus need specific approaches, tailored to the local public health situation.

## Author Contribution Statement

FBS, AB, SSP, DA, HG, PWY, AAN, RCRAP, and ADW contributed equally to the process of drafting the protocol. Both RCRAP and ADW contributed equally for developing the search strategy and running the search. FBS, RCRAP, and HG selected studies for inclusion and extracting data. The risk of bias was assessed by FBS and HG. All authors carried out and interpreted the analysis. FBS, PWY, and HG wrote the manuscript. All authors have read and approved the manuscript. 
